# Stable feature selection and classification algorithms for multiclass microarray data

**DOI:** 10.1186/1745-6150-7-33

**Published:** 2012-10-02

**Authors:** Sebastian Student, Krzysztof Fujarewicz

**Affiliations:** 1 Institute of Automatic Control, Silesian University of Technology, Akademicka 16, 44-100 Gliwice, Poland

## Abstract

**Background:**

Recent studies suggest that gene expression profiles are a promising alternative for clinical cancer classification. One major problem in applying DNA microarrays for classification is the dimension of obtained data sets. In this paper we propose a multiclass gene selection method based on Partial Least Squares (PLS) for selecting genes for classification. The new idea is to solve multiclass selection problem with the PLS method and decomposition to a set of two-class sub-problems: one versus rest (OvR) and one versus one (OvO). We use OvR and OvO two-class decomposition for other recently published gene selection method. Ranked gene lists are highly unstable in the sense that a small change of the data set often leads to big changes in the obtained ordered lists. In this paper, we take a look at the assessment of stability of the proposed methods. We use the linear support vector machines (SVM) technique in different variants: one versus one, one versus rest, multiclass SVM (MSVM) and the linear discriminant analysis (LDA) as a classifier. We use balanced bootstrap to estimate the prediction error and to test the variability of the obtained ordered lists.

**Results:**

This paper focuses on effective identification of informative genes. As a result, a new strategy to find a small subset of significant genes is designed. Our results on real multiclass cancer data show that our method has a very high accuracy rate for different combinations of classification methods, giving concurrently very stable feature rankings.

**Conclusions:**

This paper shows that the proposed strategies can improve the performance of selected gene sets substantially. OvR and OvO techniques applied to existing gene selection methods improve results as well. The presented method allows to obtain a more reliable classifier with less classifier error. In the same time the method generates more stable ordered feature lists in comparison with existing methods.

**Reviewers:**

This article was reviewed by Prof Marek Kimmel, Dr Hans Binder (nominated by Dr Tomasz Lipniacki) and Dr Yuriy Gusev

## Background

Recent studies suggest that gene expression profiles may represent a promising alternative for clinical cancer classification. Molecular-based approaches have opened the possibility of investigating the activity of thousands of genes simultaneously and can be used to find genes involved in neoplasia. A well known problem in applying microarrays in classification problem is dimension of obtained datasets. In work [1] authors listed three main sources of the instability of feature selection in biomarker discovery: choosing selection algorithms without considering stability, the existence of multiple sets of true markers and small number of samples. They suggested that the problem of small number of samples in high dimensional feature space is the most difficult one in biomarker discovery. Other authors indicate a technical problems, like post-hybridization washing [2], or chip-specific systematic variations on the raw intensity level [3], which can cause errors in computed expression levels and may have a big influence on the instability of feature selection. In [4] authors denoted the same problems not only for microarray data, but also for proteomic mass spectometry data. Traditional statistical methodology for classification does not work well when there are more variables than samples. Thus, methods able to cope with the high dimensionality of the data are needed. In this paper we focus on multiclass feature selection and classification problem, which are intrinsically more difficult than their binary counterparts [5]. Gene selection for a classifier is a very important problem. Over the past few years many algorithms were proposed to solve this problem. However, most of the studies are designed for dimension reduction in two-class problems and only a few of them involve multiclass cases. In [6,7] authors underline, that selection of informative features for a classifier is a crucial and delicate task. The optimal selection of informative genes for multiclass analysis is still an open problem. We propose a gene selection method based on Partial Least Squares (PLS) [8,9]. Then we compare the results with the multiclass gene selection method proposed in [10], Recursive Feature Elimination (RFE) method [7] and the classical t-statistics.

The standard way to use the PLS algorithm is for feature extraction only and not for selecting significant features. Here, we use this method for gene ranking. In [8] it has been shown how to use the PLS method for multiclass feature extraction. Also in [11] the author considers a PLS-based method to gene selection, but for 2-class data only. The new idea is to use the PLS for multiclass feature selection. A well known method of solving the multiclass feature selection problem is to take into consideration ‘all classes at once’.

We propose a new method based on decomposition of a multiclass feature selection problem into a set of two-class problems that are used in one versus rest (OvR) and one versus one (OvO) techniques.

An important aspect of feature selection methods is the stability of obtained ordered lists [1,12]. In [1] we can find a review that summarizes some stable feature selection methods and a big range of stability measures. Authors have noted that stable feature selection is a very important problem, and they have suggested to pay more attention on it.

In literature [13] most of the feature selection and classification methods are compared based on the accuracy rate only. In general we can define the accuracy rate, as the percentage of correctly classified probes among all probes (in most cases in the validation set). It is very difficult to evaluate the methods only by the small differences in accuracy rate. In this paper we use the stability criterion and accuracy rate to clearly compare different gene ranking methods. By better stability, we mean less variability of the ranked lists obtained with the same method, but with slightly modified datasets. The stability problem of gene lists is very important for their validation by biological methods and for the clinical applicability of molecular markers. For example, for long gene lists, experimentalists will test only the most important genes, in this case the top-ranked genes.

## Methods

## Symbols and abbreviations

*l* — number of samples; *m* — number of genes; *g* — number of selected genes; *L* — list of selected genes; *K* — number of classes; *B* — number of bootstrap samples; PLS — Partial least squares regression method; *s* — number of PLS components; *w* — PLS weight vector; SIMPLS, NIPALS — names of two used PLS algorithms; PLS+MCLASS — PLS based multiclass gene selection method with ‘all classes at once’ approach; PLS+OvO — PLS based multiclass gene selection method with ‘one versus one’ decomposition; PLS+OvR — PLS based multiclass gene selection method with ‘one versus rest’ decomposition; GS — gene selection method proposed in [10]; RFE — Recursive Feature Elimination gene selection method; *s*_1_, *s*_2_ — stability score indicators; SVM OvO, SVM OvR, MSVM — support vector machines based classification methods; LDA — linear discriminant analysis classification method;

## Bootstrap resampling

We use bootstrap technique [14] which has good performance for relatively small sample classification problems [15]. In literature we can find many publication using bootstrap resampling for genomic data [16-21]. Of course, the best way to test classification and gene selection methods is to use an independent dataset. However, without such a dataset, the resampling approach is one of the best choice. For example in [16] we can see that resampling technique is useful for microarray data analysis, and the results can be validated by qPCR analysis with an extra and independent set of samples not used in the main analysis. In our opinion the main problem in case of microarray results validation is to find proper gene selection method for analyzed data.

Let us consider a dataset of size *l*, where ***X***=(*x*_1_*x*_2_,…,*x*_*l*_) is the input matrix and ***Y***=(*y*_1_*y*_2_,…,*y*_*l*_) is the response (class labels). For multiclass problem *y*_*i*_∈{1,2,…,*K*}, where *K* is the number of classes. The bootstrap sample is a random sample with replacement of the observations and has the same size as our original dataset. The probes that appear in a bootstrap sample constitute a training dataset. The rest of observations is used as a test dataset. This is done *B* times to produce *B* bootstrap samples. To divide our samples into training and test datasets we use the balanced bootstrap method [22,23]. The balanced bootstrap is a modified version of the bootstrap method that can reduce error variance and bias over the standard bootstrap method. This method forces each observation to occur in total *B* times in the collection of *B* bootstrap samples. This does not mean that all samples should occur in every bootstrap sample, because the first observation can occur for example twice in the first bootstrap sample and not at all in the second. We can do this by constructing a set with *B* copies of all *l* observations and then permuting the obtained set. Every *l*-element successive subset is one bootstrap sample.

The bootstrap resampling is computationally costly. We implemented it on a computer cluster using the MatlabMPI toolbox for parallel computation. The most important parameter for the bootstrap resampling technique is the number of resampling iterations *B*. We must find the compromise between analysis time and accuracy of predicted parameters. In our cases we use 500 resampling iterations of all stages of the classifier construction (i.e. gene preselection, gene selection and classifier learning). We did not observed significant changes in the results for all used datasets, after increasing the number of iterations. Of course, the necessary iterations number can change after changing the dataset and depends especially on the number of probes. We generated 500 bootstrap samples only once to reduce the variability of results for all tested methods. The distribution of the misclassification rate obtained during all bootstrap runs was used to estimate the 95% confidence interval. The accuracy of the classifier and the confidence interval were calculated for subsets of first genes on the lists up to 30 genes.

### Prediction error estimation

To estimate the prediction error (accuracy) we used the.632+ estimator [24]. The.632+ estimator described by Efron provides protection of overfitting, especially important for methods like SVM, where the resubstitution error is very small. In extreme case, when the resubstitution error is very small, and much smaller than the test error, the.632 [25] estimator provides too optimistic estimates for the true error. In this situation the.632+ estimator takes more weight to the test error part, than the.632 estimator. The detailed description for the.632+ estimator is given in the Appendix.

## PLS-based feature selection method

In this section we propose a new method for selecting the most significant genes. It is based on partial least squares regression (PLSR) [26]. There are some other regression methods like Lasso method [27] or ridge regression [28]. It was shown that PLS method outperforms Lasso method in terms of identifying relevant predictors [29]. We also do not use the ridge regression, where it is a problem with estimation the ridge parameter. PLSR method is well known as a method for feature extraction [8,30,31], but its application for selecting significant genes is less evident. PLS feature extraction method can be used for significance analysis of gene expression data [32,33]. The authors of [34] used jackknife of PLS components to interpret the importance of the variables (genes) for the PLS model. When we use the feature extraction techniques like those based on projection (e.g. principal component analysis) or compression (e.g. based on information theory), we use all genes in our model (with different weights), and the accuracy of the classifier is estimated for all of the genes. In contrast to feature extraction, feature selection techniques do not alter the original representation of the variables, but only select their subset. Feature selection is very important for biomarker discovery, specifically for RT-PCR experiment and leads to new knowledge about the biology of the disease. In that case, the genes selected are more important than the classifier used. In Boulesteix [31,35], the PLS connection to other statistical methods is described. Boulesteix proved that in case of the data matrix scaled to unit variance and two-class classification the lists of genes obtained with ordered squared weight vector ***w***^2^ from the first PLS component is of the same order as from F-statistics. It is equivalent to the t-test with equal variance and also with the BSS/WSS-statistics, where BSS denotes the between-group sum of squares and WSS the within-group sum of squares. In our comparison we did not scale the data to unit variance, but only centered the data. Boulesteix and Strimmer [35] describe and refer the connection of PLS to gene selection based on “variable importance in projection” (VIP) indicator proposed by Musumarra et al. [36], which indicates the importance of genes in the used PLS latent components. Musumarra et al. described the PLS method as dimension reduction method and used the weight vectors to order genes in term of their relevance for classification problem. The main difference between our approach and VIP indicator is that in VIP method the latent components for classifier and the weight vector are used only for measure of the importance of each gene in PLS model.

In this paper we use the weight vector obtained from the PLS method to select the most important genes.

PLS aims at finding uncorrelated linear transformations of the original input features which have high covariance with the response features. Based on these latent components, PLS predicts response features (the task of regression) and reconstructs an original dataset matrix (the task of data modeling) at the same time. For dataset matrix ***X*** of size *l*×*m* with *l* probes and *m* genes we denote the *l*×1 vector of response value ***y***. The PLS components ***t***_*i*_*i*=1,…,*s*are constructed to maximize the objective criterion based on the sample covariance between ***y*** and linear combination of genes (PLS components) ***t***=***Xw***. We search the weight vector ***w***sequentially, to satisfy the following criterion

(1) $$w_{i}=\underset{w^{T}w=1}{\mathrm{argmax}}\;{\text{cov}}^{2}(Xw,y),$$

subject to the orthogonality constraint

(2) $$\begin{array}{rll} w_{i}^{T}S_{X}w_{j} & =0\;1\leq i<j\; & \;\\ S & =X^{\prime }X.\; \end{array}$$

This criterion is the mostly used in literature as general description for PLS method. In case of multiclass categorical data this criterion can be simplified as mentioned in [37] and maximize var(***Xw***) Cor^2^(***XwY***). To derive components (named “latent variables” or scores), ***t***_*i*_(*i*=1,…,*s*), the PLS decomposes ***X***and ***y*** to produce a bilinear representation of the data [38]

(3) $$\begin{array}{lll} X & =t_{1}p_{1}^{T}+t_{2}p_{2}^{T}+\ldots +t_{s}p_{s}^{T}+E\;\\ y & =t_{1}q_{1}+t_{2}q_{2}+\ldots +t_{s}q_{s}+f,\; & \; \end{array}$$

where ***p***_***i***_ are loadings, *q*_*i*_ are scalars and ***Ef*** are residuals. The idea of PLS is to estimate loadings and scores by a regression. The PLS fits a sequence of bilinear models by least squares. At every step *i*(*i*=1,…,*s*) vector ***w***_*i*_ is estimated to obtain the PLS component that has maximal sample covariance with the response variable ***y***. Each component ***t***_*i*_is uncorrelated with all previously constructed components. There are two main PLS algorithms described in literature: NIPALS algorithm [39] and SIMPLS algorithm [40]. The SIMPLS algorithm, is different from NIPALS in two important ways: first, successive *t*_*i*_components are calculated explicitly as linear combinations of X and second, X is not deflated in each iteration. The SIMPLS algorithm will be assessed in accordance with the criteria eq. (1). In NIPALS the first PLS component ***t***_1_ is obtained on the basis of the covariance between ***X***and ***y***, and is qual to the first component of SIMPLS algorithm. Component ***t***_*i*_(*i*=2,…,*s*), is computed using the residuals of ***X***and ***y*** from the previous step, which account for the variations left by the previous components. Maximal number of components *s* is equal to the rank of ***X***.

As we say before the weight vector from SIMPLS algorithm sometimes referred to ***r*** is applied to the original ***X*** matrix and De Jong [40] showed that we can calculate the weights ***r***directly from the NIPALS algorithm

(4) $$r_{i}=w_{i}{\left(p_{i}^{\prime }w_{i}\right)}^{\left(-1\right)},$$

where ***p***_*i*_ are the loading and ***w***_*i*_are the weight vector for i-th component of NIPALS algorithm.

**Figure 1:**
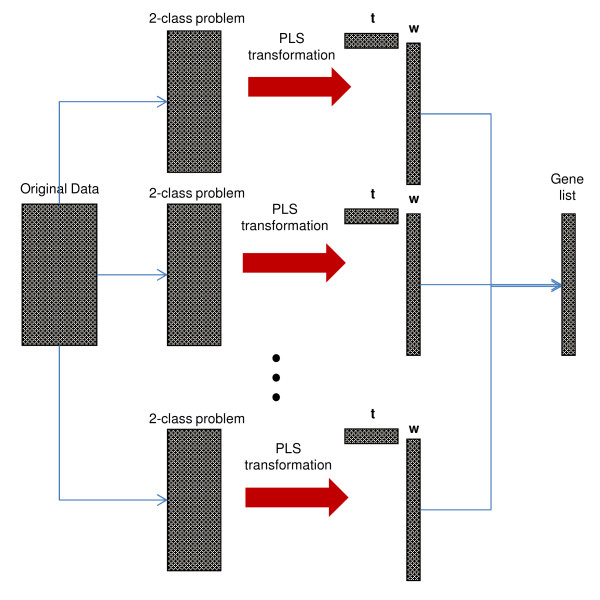
PLS based gene selection method with two-class decomposition technique.

De Jong proved in [40] that for univariate response the score vectors ***t***_***i***_(*i*=*i*,…,*s*) for NIPALS and SIMPLS algorithms are the same. In contrast to score vectors, the weight vectors ***w***_***i***_ and ***r***_***i***_ for NIPALS and SIMPLS respectively are different for *i*>1. This phenomenon is a consequence of different method to compute the weights vectors. The ***w***_***i***_vectors in NIPALS procedure are calculated with deflated data matrices ***X***_***i***_ and ***Y***_***i***_ in each iteration, and the weights ***r***_***i***_are obtained without the deflation step in SIMPLS algorithm. For this reason in this paper, we use the weight vectors ***w*** and ***r***from both algorithms to determine the ranked list. In our method the sum of the $w_{i}^{2}$ over the *s* PLS components presents the gene importance vector and the “best genes” have the highest values in this vector. First *g* genes with the highest value in the gene importance vector are selected for the classifier. To test the optimal number of components we use the first squared weight vector and the sum of squared weight vectors from first 5 and 10 components. The standard way to use PLS for a multiclass data, is to search for the best direction for maximization of the covariance between responses with all classes and linear combination of genes. As we mentioned before, we compare our method based on decomposition of a multi-class feature selection problem into a set of two-class problems with a well known ‘all classes at once’ technique. For each two-class selections “best genes” are selected and one ranked gene list is constructed as follows: genes with the highest weight in all two-class selections are located at the top of the list, then genes with the second highest weights, and so on. We must underline, that ***y***for two-class selections is coded as a vector with value 1 for the first class and −1 for the second class. For the ‘all classes at once’ technique ***y***is a matrix with *N* rows and the number of columns is equal to the number of classes. In each row a class label has a value of 1 and −1. For our needs we introduce the notation PLS+MCLASS for ‘all classes at once’ technique and similarly PLS+OvO, PLS+OvR for two-class decomposition of the multiclass feature selection problem. On the Figure 1 we show the principals and essentials of the introduced method.

## Stability analysis for ordered gene lists

In [6,41] authors have used resampling technique for testing the significance of the obtained results of microarray analysis. They have examined the influence of sample class label permutations and selection of exact number of randomly selected features on the classification accuracy. We can find in literature various applications of bootstrap technique for example to assess the stability of the cell lines cluster dendrogram in unsupervised microarray analysis [42]. In our article we use bootstrap resampling to examine the stability of obtained gene lists. By stability of an obtained gene list we understand similarity between lists from the same experiment, but with a slightly changed data set. To show the distance between different gene selection methods we use a method based on bootstrap resampling. This approach is based on the comparison of sets consisting of a fixed number of the top *g* genes. In this framework we consider the list ***L*** with first *g* top-genes obtained from the entire dataset and lists ***L***_*b*_;*b*=1,2,…,*B*obtained from every *b* of *B* bootstrap iterations.

In this paper we assess stability in two ways. The first one is to calculate stability indices. In this case we have used Percentage of Overlapping Genes (POG) criterion [43] and modified POG indicator. The POG criterion takes into account only the content of gene lists, and ignores the gene order. The modified POG indicator does not ignore the gene order on compared lists. Both indicators are detailed described in the Appendix.

The second one is to visualize how obtained gene lists are stable by looking at descriptive plots. In the next section we introduce the detailed description of the stability plots used in this article.

### Stability plots

To visualize the stability of the ordered gene lists we plot the boxplots of rank for each gene in the list ***L*** against ranks in all *b* bootstrap iteration lists ***L***_*b*_;*b*=1,2,…,*B*. We set the limit to determine which points are extreme to the rank out of the *g* gene list. Another way to visualize gene lists stability is to plot a so-called Bootstrap Based Feature Ranking (BBFR) plot [44]. The BBFR score, in opposite to indices *s*_1_and *s*_2_, is calculated separately for each gene. The BBFR score for the gene number *j* is defined as

(5) $$Q_{j}=\frac{\overset{B}{\underset{b=1}{\sum }}r_{\text{bj}}}{\mathrm{Bg}},$$

where *r*_*bj*_ is the *rank* of the *j*-th gene in *b*-th bootstrap iteration

(6) $$r_{\text{bj}}=g-u_{\text{bj}}+1$$

for the top-scored gene *r*_*bj*_=*g*.

The maximum possible value of the *Q*_*j*_score is 1. It means that one gene was top-ranked in all *B* bootstrap iterations. The score *Q*_*j*_ takes into account the rank *r*_*bj*_of *j*-th gene in all *B* bootstrap iterations.

The modified BBFR score $Q_{j}^{\prime }$ takes into account only the presence of the gene *j* in the lists *L*_*b*_;*b*=1,2,…,*B*

(7) $$Q_{j}^{\prime }=\frac{\overset{B}{\underset{b=1}{\sum }}I(u_{\text{bj}}\leq g)}{B}.$$

The maximum possible value of the $Q_{j}^{\prime }$ score is 1. The 0 value indicates genes not included on the gene lists in all bootstrap iterations.

Both *Q*_*j*_and $Q_{j}^{\prime }$ indices are sorted and plotted in descending order. In this paper we use only the second ranking plot $Q_{j}^{\prime }$. In the ideal case (when gene lists are perfectly reproducible) the $Q_{j}^{\prime }$ plot reaches a value of 1 for the first *g* genes and 0 for the rest.

## Datasets

In our study we chose three publicly available multiclass microarray datasets. The first is the LUNG dataset published by [45]. It consists of 254 samples of 4 subtypes of lung carcinomas and normal samples. Samples were normalized by RMA and GA annotation [46]. Each sample has 8359 gene expression levels after re-annotation. The data is available at http://www.broadinstitute.org/mpr/lung/. The second is the MLL dataset published by [47]. It consists of 72 samples of 3 subtypes of leukemia cancer classes. Samples was normalized by RMA and GA annotation [46]. Each sample has 8359 gene expression levels after re-annotation. The data is available at http://www.broadinstitute.org/cgi-bin/cancer/publications/pub_paper.cgi?mode=view&paper_id=63. The third is the SRBCT dataset published by [48]. It consists of 83 samples of 4 subtypes of small, round blue cell tumors. Each sample has 2308 gene expression levels. The data is available at http://www.biomedcentral.com/content/supplementary/1471-2105-7-228-s4.tgz[10]. The results for the LUNG dataset are presented in the main body of this paper, and the results for MLL and SRCT datasets are presented in the Appendix section.

## Results and Discussion

We chose three multiclass microarray datasets (detailed described in the Datasets section) for our experiments.

**Figure 2:**
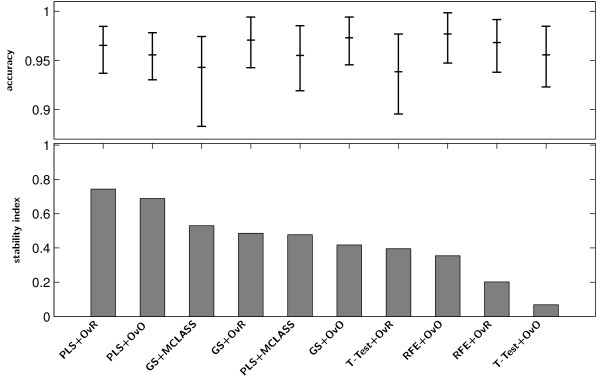
**Stability index*s***_***2***_**(bar chart) and accuracy of classification (dot chart) with the 95% confidence interval of the best classifier on the tested feature selection methods for LUNG data.**

For the numerical experiment we use SVM method classification method in three variants OvO, OvR, MSVM and LDA method. These methods are common used in microarray classification problems [49-51]. We demonstrate the usefulness of the proposed methodology to select significant genes with decomposition technique and the PLS method. All methods: PLS+OvO, PLS+OvR and PLS+MCLASS were tested and compared with other methods. As it has been mentioned before, we executed 500 bootstrap iterations for each method. Because the most important task is to find a small number of informative genes, we classify this data in every bootstrap iteration for diverse number of best genes up to 30 genes. In Tables 1, 2 and 3 (Tables are available in the Appendix) we collect all results for all tested methods. For all plots we use the classifier with the best classification rate chosen separately for all tested method. The PLS algorithm and the number of PLS components were chosen with respect to the best accuracy rate criterion. In most cases the ***r*** vector calculated from SIMPLS method was better than the vector ***w***calculated from NIPALS algorithm for more than one component. Only for PLS+MCLASS method the accuracy rate is higher when we use more than 1 PLS component. In our study we also applied a method searching for the optimal number of components based on leave one out classification error on training samples and the SVM classifier (results not showed here). In general the results for classification accuracy rate were not significantly better and in some cases even worse. In all tables we bolded the best accuracy rate for tested classification methods and variants of PLS method (algorithm and number of PLS components). In the last columns we show the standard deviations values for the best classifier. The comparison of accuracy rate and stability index *s*_2_for all tested datasets proves the advantage of the PLS method (Figure 2). In all cases stability index *s*_2_ for the PLS method with decomposition technique is higher than the score for the PLS+MCLASS method. Only for the LUNG dataset the stability index for decomposition version of the GS method is lower than with the GS+MCLASS method (Figure 2). However, in this case, the accuracy rate for the GS+MCLASS was about 3% lower than for the GS+OvO method. Consequently, looking at all the classification accuracies and the 95% confidence interval as shown in Tables 1, 2 and 3, one general conclusion is that there are no significant differences between best gene selection methods. Typically, our methods outperform the other methods when we compare the stability index. Another conclusion is that more components spoil the stability of obtained genes lists and the classification error is not significantly smaller.

**Figure 3:**
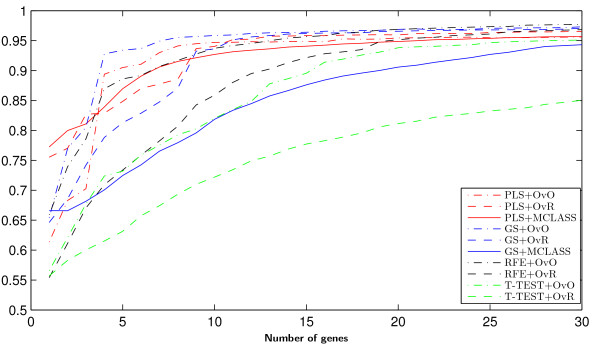
Accuracy of classification obtained by successive gene set reduction selected with all feature selection methods of the best classifier for LUNG data.

On Figure 3 we can see how many genes we need to obtain good prediction. For the arbitrary changed number of features selected we built the model and estimate the accuracy rate. We do not use the accuracy rate to estimate the number of selected genes as in backward elimination features selection. When we compare the results for different datasets (for example Figure 3, and Figure 8, Figure 9 from Appendix), we can see, that in all cases the two class decomposition based gene selection methods are better for different number of selected genes, when we consider the accuracy rate into account. However, we can see that there are big differences between used methods, especially, when we use a very small number of genes. We also observe, slight different accuracy results for the different data sets and selected gene number, especially in the dynamics of accuracy rate for increased number of genes. This means, that the number of selected genes depends on dataset used and is important for distinguish the best gene selection method (for example comparison of Accuracy rate results for LUNG data on Figure 3 and for MLL data set Figure 8 from Appendix).

In all tested datasets the 30 genes were sufficient enough to obtain a high accuracy rate. In all datasets the decomposition variants of the GS method outperform the GS+MCLASS method. The PLS+OvO and PLS+OvR methods perform at least comparably well and for the MLL dataset the accuracy rate was higher for different number of selected genes.

**Figure 4:**
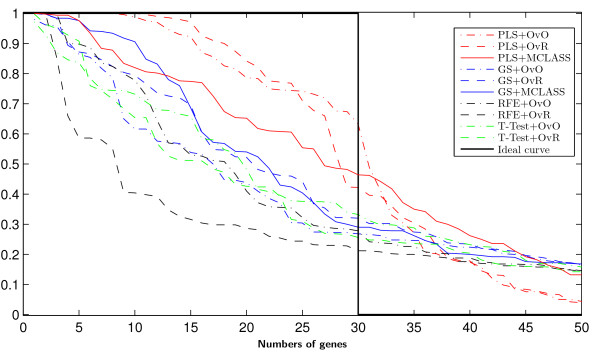
**Results of bootstrap-based feature ranking (BBFR) for the first 50 genes for LUNG data.** In the ideal case (when gene lists are perfectly reproducible) the BBFR score reaches a value of 1 for the first selected genes and 0 for the rest (black curve).

**Figure 5:**
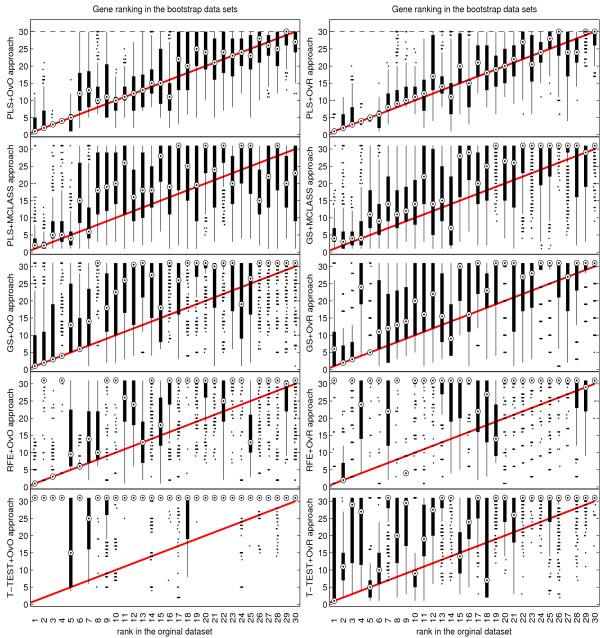
Comparison of rank boxplots in the bootstrap samples against rank in the original data set on all tested methods for LUNG data.

The bootstrap-based feature ranking (BBFR) is computed for a list of 30 genes. The BBFR ranking (Figure 4) and the boxplots of rank for each gene in the bootstrap lists versus the whole dataset gene list (Figure 5) confirm the advantage of the proposed gene selection method. For all datasets only the BBFR curves for PLS+OvO and PLS+OVR are very close to ideal curve. This means that the same genes are reselected frequently in most bootstrap iterations. In Tables 1, 2 and 3 we can see, that for the PLS method we observe the smallest number of all genes selected in all bootstrap iteration 30-genes lists (reselected genes column). That means, that the reproducibility of the PLS method is very high in contrast to other methods, where we observe more than one hundred genes more. Our conclusion for different number of selected genes is confirmed by the boxplots in Figure 5 for tested methods. The figures illustrate how close the bootstrap based feature ranking is to the ranking obtained from the whole datasets for the first 30 genes. The red line indicate the ideal case. The best reproducibility is found with the PLS method. The worst reproducibility is found for the classical T-TEST and RFE method.

## Conclusions

In this paper we proposed a new PLS-based method to select significant genes. Our results have shown that this gene selection method gives very good accuracy rate and stability of obtained gene lists. The principal of PLS is based on the maximization of the covariance criterion, which can lead to good generalization ability and stability. In our opinion this is a reason of the good results obtained with PLS method. Another important result is the fact that it is more effective to solve a multiclass feature selection by splitting it into a set of two-class problems and merging the results in one gene list. The explanation for these result can be the difference between used methods: the idea of MCLASS approach is to look for genes able to distinguish between all classes simultaneously. Such genes are more difficult to find, and they can have smaller discriminatory power. This problem do not exist in the decomposed multiclass problem for OvO and OvR approaches. From the methodological side we suppose, that the MCLASS multiclass feature selection methods are not so good developed, as the 2-class methods, and this fact can be the explanation for our results. The comparison to other feature selection methods shows that the gene lists stability index is the highest for PLS with OvR and OvO techniques. In two cases the stability index is slightly better for PLS+MCLASS method with one PLS component, but the accuracy rate for this method is significantly worse. All other methods indicated much worse stability of obtained gene lists. We can observe that using the GS method with 2-class decomposition technique improves the accuracy rate and with two of the datasets gene list stability increased as well. Another advantage of the 2-class decomposition technique for gene selection methods is easy interpretation of the results by biologists. In all cases the ‘all classes at once’ technique of PLS and GS methods achieves worse classification accuracy than their 2-class versions. The presented method makes it possible to obtain more stable gene selection lists and a more reliable classifier with less classifier error. We show that accuracy rate assessing accompanied with the gene stability analysis gives more reliable evaluation of various gene selection methods. Of course our methods can be applicable also to other high dimensional data where we consider classification problems such as protein microarrays, DNA copy number variation, exome profiling and RNAseq. In all cases, where the dataset has much more features than observations it is recommended to take into consideration the accuracy rate, but also the stability score.

## Appendix

### Stability indices

In general there are two different approaches to measure the stability of gene lists. The first approach takes into account only the content of gene lists, and ignores the gene order. The second one does not ignore the gene order on compared lists.

One of the most frequently used criteria is the Percentage of Overlapping Genes (POG) [43], belonging to first class of stability measures. In the simplest case it measures the similarity between two lists *L*_1_ and *L*_2_ of the size *g*. Let *k* be the size of the intersection of *L*_1_and *L*_2_. Then POG is defined as *s*_1_=*k*/*g*.

POG criterion may be extended in such a way that it measures the similarity between the list ***L*** and lists ***L***_*b*_;*b*=1,2,…,*B*. Let *u*_*j*_ be the placement of the *j*-th gene in the list ***L***. For the top-scored gene *u*_*j*_=1. Similarly, *u*_*bj*_ is the placement of the *j*-th gene in the list ***L***_*b*_. The POG is calculated as

(8) $$s_{1}=\overset{B}{\underset{b=1}{\sum }}\frac{\overset{g}{\underset{j=1}{\sum }}I(u_{j}\leq g\wedge u_{\text{bj}}\leq g)}{\mathrm{Bg}},$$

where *I* denotes the indicator function

(9) $$I(A)=\left\{\begin{array}{c} 1\;\;\text{if}\;\;A=\mathrm{true}\\ 0\;\;\text{if}\;\;A=\mathrm{false} \end{array}\right)$$

We introduce the modified relative *s*_2_score to estimate the similarity between all lists

(10) $$s_{2}=1-\overset{B}{\underset{b=1}{\sum }}\overset{g}{\underset{j=1}{\sum }}\frac{\left|u_{j}-u_{\text{bj}}\right|}{\mathrm{Bg}(g+1)/2}.$$

In opposite to previous indicator *s*_1_, it does not ignore the rank of the selected genes within the considered subset, hence it belongs to the second mentioned class of stability measures. The value for the gene that is out of ***L***_*b*_ is set to *g* + 1. The value of functions *s*_1_ and *s*_2_ is scaled to the interval 〈0,1〉 and the higher value indicates better stability of the obtained gene list.

Another indicator used to estimate the stability of an obtained gene list is given by the number of genes that were selected at least one time in all bootstrap samples. The best value is *g* and the worst is *Max*(*G*,*Bg*) where *G* is the number of all genes. This approach is equal to the number of genes with a non-zero score in the Bootstrap Based Feature Ranking (BBFR) (described in the next section).

## Prediction error estimation

To estimate the prediction error we use the.632+ estimator [24]. First we must define the prediction model as $\hat{f}(X)$ which can be estimated from a training sample. The loss function for measuring errors between ***Y*** and $\hat{f}(X)$ we can describe as $L(Y,\hat{f}(X))$. This function returns 0 if response ***Y***equals predicted value $\hat{f}(X)$ and 1 otherwise. Now we can define the resubstitution error

(11) $${\hat{\mathrm{Err}}}_{\text{resub}}=\frac{1}{l}\overset{l}{\underset{i=1}{\sum }}L\left(y_{i},\hat{f}(x_{i})\right),$$

where $\hat{f}(x_{i})$ is the predicted value at *x*_*i*_of the whole dataset. This predictor can make overfitted predictions and the estimated error rate will be downward biased. It demonstrates why we obtain error estimator for test data sets in the form

(12) $${\hat{\mathrm{Err}}}_{\text{test}}=\frac{1}{B}\overset{B}{\underset{b=1}{\sum }}\frac{1}{\left|C_{b}\right|}\underset{i\in |C_{b}|}{\sum }L\left(y_{i},{\hat{f}}^{*b}(x_{i})\right).$$

The model trained on a training set will be tested on other samples and not used to fit the model. This provides protection against overfitting. As we have mentioned before, we compute the error rate for *B* sets *C*_*b*_ containing samples that do not appear in *b*-th bootstrap sample and |*C*_*b*_| is a number of such samples. This estimator will overestimate the true prediction error, and when the test set is small it can have high variance [15]. To resolve this problem we use the.632+ estimator. This is a modified version of the.632 estimator to avoid downward bias in overfitting case of our classifier. Define *γ*to be the error rate of our prediction rule if the inputs and class labels are independent. Let $\hat{p_{k}}$ be the observed proportion of responses *y*_*i*_equal *k* and let $\hat{q_{k}}$ be the proportion of predictions $\hat{f}(x_{i})$ equal *k*, where *k* is the class label of *K* class. Then

(13) $$\hat{\gamma }=\overset{K}{\underset{k=1}{\sum }}\hat{p_{k}}\left(1-\hat{q_{k}}\right).$$

The relative overfitting rate is

(14) $$\hat{R}=\frac{{\hat{\mathrm{Err}}}_{\text{test}}-{\hat{\mathrm{Err}}}_{\text{resub}}}{\hat{\gamma }-{\hat{\mathrm{Err}}}_{\text{resub}}}.$$

Now we can define the.632+ estimator by

(15) $${\hat{\mathrm{Err}}}_{\left(.632+\right)}=(1-ŵ){\hat{\mathrm{Err}}}_{\text{resub}}+ŵ{\hat{\mathrm{Err}}}_{\text{test}},$$

(16) $$ŵ=\frac{0.632}{1-0.368\hat{R}}.$$

When there is no overfitting problem the.632+ estimator is equal to the.632 estimator

(17) $${\hat{\mathrm{Err}}}_{\left(.632+\right)}=0.368{\hat{\mathrm{Err}}}_{\text{resub}}+0.632{\hat{\mathrm{Err}}}_{\text{test}}.$$

### Tables

Table 1 The bootstrap based classification accuracies, stability index and number of reselected genes in all bootstrap samples of the SVM-classifier and the LDA-classifier based on all tested gene selection methods, on the LUNG dataset.

**Table 1:** The bootstrap based classification accuracies, stability index and number of reselected genes in all bootstrap samples of the SVM-classifier and the LDA-classifier based on all tested gene selection methods, on the LUNG dataset

**method**			**stability index**		**Reselected genes**	**Classification method**				**Best result**		
						**SVM OvO**	**SVM OvR**	**MSVM**	**LDA**			
			***s***_**1**_	***s***_**2**_		**acc**	**acc**	**acc**	**acc**	**acc**	**accL**	**accH**
SIMPLS	OvO	1 comp.	0.86	0.69	84	0.954	0.946	0.951	**0.956**	0.956	0.930	0.978
	OvR	1 comp.	0.86	0.74	78	0.950	0.945	0.947	**0.965**	0.965	0.937	0.985
	MCLASS	1 comp.	0.88	0.79	70	0.917	0.882	0.892	0.895	0.917	0.868	0.956
	OvO	5 comp.	0.60	0.51	186	0.950	0.928	0.944	0.966	0.966	0.933	0.986
	OvR	5 comp.	0.71	0.61	170	0.958	0.946	0.953	0.963	0.963	0.940	0.986
	MCLASS	5 comp.	0.75	0.58	103	0.953	0.945	0.949	0.917	0.953	0.920	0.979
	OvO	10 comp.	0.58	0.44	214	0.949	0.928	0.941	0.946	0.949	0.913	0.979
	OvR	10 comp.	0.63	0.49	228	0.955	0.944	0.950	0.961	0.961	0.931	0.983
	MCLASS	10 comp.	0.72	0.48	128	**0.955**	0.949	0.950	0.943	0.955	0.919	0.985
NIPALS	OvO	1 comp.	0.86	0.69	84	0.954	0.946	0.951	0.956	0.956	0.930	0.978
	OvR	1 comp.	0.86	0.74	78	0.950	0.945	0.947	0.965	0.965	0.937	0.985
	MCLASS	1 comp.	0.88	0.79	70	0.917	0.882	0.892	0.895	0.917	0.868	0.956
	OvO	5 comp.	0.68	0.60	151	0.949	0.932	0.943	0.956	0.956	0.924	0.980
	OvR	5 comp.	0.76	0.62	126	0.954	0.946	0.949	0.954	0.954	0.923	0.980
	MCLASS	5 comp.	0.74	0.62	111	0.949	0.940	0.944	0.930	0.949	0.907	0.979
	OvO	10 comp.	0.66	0.46	143	0.946	0.920	0.936	0.927	0.946	0.907	0.978
	OvR	10 comp.	0.71	0.51	134	0.950	0.938	0.944	0.936	0.950	0.910	0.979
	MCLASS	10 comp.	0.73	0.55	121	0.951	0.944	0.947	0.911	0.951	0.912	0.979
RFE	OvO		0.44	0.35	487	0.962	0.953	0.961	**0.977**	0.977	0.947	0.999
	OvR		0.30	0.20	808	0.965	0.961	0.964	**0.968**	0.968	0.938	0.992
T-TEST	OvO		0.09	0.07	333	**0.956**	0.939	0.951	0.951	0.956	0.923	0.985
	OvR		0.47	0.40	624	**0.939**	0.925	0.934	0.850	0.939	0.896	0.977
GS	OvO		0.52	0.42	453	0.962	0.944	0.957	**0.973**	0.973	0.946	0.994
	OvR		0.62	0.49	305	0.964	0.953	0.961	**0.971**	0.971	0.943	0.994
	MCLASS		0.65	0.53	243	0.935	0.900	0.921	**0.943**	0.943	0.883	0.974

^a^The number of selected genes is set to 30 In last 3 columns is the best accuracy rate together with their bootstrap based standard deviations (accL,accH). The number of reselected genes is the sum of non zero bootstrap-based feature ranked genes (BBFR).

Table 2 The bootstrap based classification accuracies, stability index and number of reselected genes in all bootstrap samples of the SVM-classifier and the LDA-classifier based on all tested gene selection methods, on the MLL dataset.

**Table 2:** The bootstrap based classification accuracies, stability index and number of reselected genes in all bootstrap samples of the SVM-classifier and the LDA-classifier based on all tested gene selection methods, on the MLL dataset

**Selection method**			**stability index**		**Reselected genes**	**Classification method**				**Best result**		
						**SVM OvO**	**SVM OvR**	**MSVM**	**LDA**			
			***s***_**1**_	***s***_**2**_		**acc**	**acc**	**acc**	**acc**	**acc**	**accL**	**accH**
SIMPLS	OvO	1 comp.	0.77	0.70	116	0.971	0.973	0.972	**0.995**	0.995	0.971	1.000
	OvR	1 comp.	0.77	0.71	114	0.973	0.974	0.972	**0.993**	0.993	0.969	1.000
	MCLASS	1 comp.	0.81	0.73	84	0.962	0.970	0.967	0.965	0.970	0.915	1.000
	OvO	5 comp.	0.64	0.50	245	0.975	0.978	0.977	0.978	0.978	0.936	1.000
	OvR	5 comp.	0.63	0.52	224	0.976	0.978	0.975	0.981	0.981	0.943	0.995
	MCLASS	5 comp.	0.66	0.59	201	0.975	0.977	0.976	**0.982**	0.982	0.943	0.995
	OvR	10 comp.	0.52	0.40	281	0.974	0.975	0.973	0.971	0.975	0.936	1.000
	MCLASS	10 comp.	0.58	0.47	236	0.975	0.976	0.974	0.972	0.976	0.936	1.000
NIPALS	OvO	1 comp.	0.77	0.70	116	0.971	0.973	0.972	0.995	0.995	0.971	1.000
	OvR	1 comp.	0.77	0.71	114	0.973	0.974	0.972	0.993	0.993	0.969	1.000
	MCLASS	1 comp.	0.81	0.73	84	0.962	0.970	0.967	0.965	0.970	0.915	1.000
	OvO	5 comp.	0.62	0.50	221	0.974	0.975	0.974	0.975	0.975	0.924	0.995
	OvR	5 comp.	0.62	0.53	198	0.975	0.976	0.975	0.977	0.977	0.938	0.995
	MCLASS	5 comp.	0.67	0.53	188	0.974	0.976	0.974	0.979	0.979	0.943	0.995
	OvO	10 comp.	0.62	0.46	211	0.973	0.975	0.974	0.963	0.975	0.928	1.000
	OvR	10 comp.	0.61	0.47	196	0.970	0.971	0.970	0.960	0.971	0.971	1.000
	MCLASS	10 comp.	0.63	0.51	204	0.971	0.973	0.971	0.957	0.973	0.973	1.000
RFE	OvO		0.58	0.48	318	0.975	0.977	0.974	**0.982**	0.982	0.940	0.995
	OvR		0.47	0.36	401	0.976	0.977	0.975	**0.988**	0.988	0.947	1.000
T-TEST	OvO		0.55	0.42	448	0.972	0.978	0.976	**0.987**	0.987	0.945	1.000
	OvR		0.59	0.49	534	0.977	0.978	0.977	**0.981**	0.981	0.935	0.995
GS	OvO		0.62	0.54	489	0.971	0.977	0.975	**0.982**	0.982	0.943	0.995
	OvR		0.64	0.50	490	0.977	0.977	0.976	**0.986**	0.986	0.947	0.995
	MCLASS		0.59	0.43	526	0.963	0.967	0.965	**0.970**	0.970	0.915	0.995
	OvO	10 comp.	0.56	0.33	268	0.969	0.972	0.971	0.960	0.972	0.919	1.000

^a^The number of selected genes is set to 30. In last 3 columns is the best accuracy rate together with their bootstrap based standard deviations (accL,accH). The number of reselected genes is the sum of non zero bootstrap-based feature ranked genes (BBFR).

Table 3 The bootstrap based classification accuracies, stability index and number of reselected genes in all bootstrap samples of the SVM-classifier and the LDA-classifier based on all tested gene selection methods, on the SRBCT dataset

**Table 3:** The bootstrap based classification accuracies, stability index and number of reselected genes in all bootstrap samples of the SVM-classifier and the LDA-classifier based on all tested gene selection methods, on the SRBCT dataset

**Selection method**			**stability index**		**Reselected genes**	**Classification method**				**Best result**		
						**SVM OvO**	**SVM OvR**	**MSVM**	**LDA**			
			***s***_**1**_	***s***_**2**_		**acc**	**acc**	**acc**	**acc**	**acc**	**accL**	**accH**
SIMPLS	OvO	1 comp.	0.71	0.60	127	0.867	**0.977**	0.976	0.941	0.977	0.913	1.000
	OvR	1 comp.	0.74	0.59	137	0.882	**0.980**	0.980	0.952	0.980	0.912	1.000
	MCLASS	1 comp.	0.58	0.39	240	0.807	0.930	0.932	0.829	0.932	0.797	1.000
	OvO	5 comp.	0.52	0.36	204	0.831	0.970	0.968	0.929	0.970	0.896	1.000
	OvR	5 comp.	0.63	0.43	173	0.867	0.981	0.979	0.964	0.981	0.926	1.000
	MCLASS	5 comp.	0.72	0.58	133	0.856	**0.979**	0.978	0.945	0.979	0.903	1.000
	OvO	10 comp.	0.40	0.25	239	0.817	0.961	0.958	0.910	0.961	0.873	1.000
	OvR	10 comp.	0.45	0.23	237	0.839	0.977	0.974	0.942	0.977	0.912	1.000
	MCLASS	10 comp.	0.63	0.41	133	0.832	0.975	0.973	0.931	0.975	0.912	1.000
NIPALS	OvO	1 comp.	0.71	0.60	127	0.867	0.977	0.976	0.941	0.977	0.913	1.000
	OvR	1 comp.	0.74	0.59	137	0.882	0.980	0.980	0.952	0.980	0.912	1.000
	MCLASS	1 comp.	0.58	0.39	240	0.807	0.930	0.932	0.829	0.932	0.797	1.000
	OvO	5 comp.	0.65	0.45	152	0.726	0.920	0.914	0.775	0.920	0.785	1.000
	OvR	5 comp.	0.68	0.47	129	0.748	0.935	0.932	0.787	0.935	0.819	1.000
	MCLASS	5 comp.	0.69	0.48	133	0.805	0.965	0.962	0.861	0.965	0.873	1.000
	OvO	10 comp.	0.66	0.43	137	0.711	0.910	0.905	0.745	0.910	0.756	1.000
	OvR	10 comp.	0.65	0.48	119	0.687	0.886	0.885	0.687	0.886	0.726	0.980
	MCLASS	10 comp.	0.67	0.46	117	0.723	0.912	0.909	0.746	0.912	0.776	1.000
RFE	OvO		0.57	0.36	262	0.925	**0.983**	0.982	0.972	0.983	0.919	1.000
	OvR		0.40	0.32	338	0.923	**0.982**	0.981	0.963	0.982	0.926	1.000
T-TEST	OvO		0.29	0.22	438	0.925	**0.971**	0.969	0.964	0.971	0.899	1.000
	OvR		0.47	0.37	498	0.929	0.972	0.971	**0.973**	0.973	0.899	1.000
GS	OvO		0.51	0.39	533	0.921	**0.969**	0.968	0.964	0.969	0.889	1.000
	OvR		0.64	0.54	367	0.927	**0.980**	0.977	0.979	0.980	0.923	1.000
	MCLASS		0.57	0.48	407	0.921	**0.959**	0.957	0.959	0.959	0.873	1.000

^a^The number of selected genes is set to 30. In last 3 columns is the best accuracy rate together with their bootstrap based standard deviations (accL,accH). The number of reselected genes is the sum of non zero bootstrap-based feature ranked genes (BBFR)

### Figures for MLL and SEBCT data

**Figure 6:**
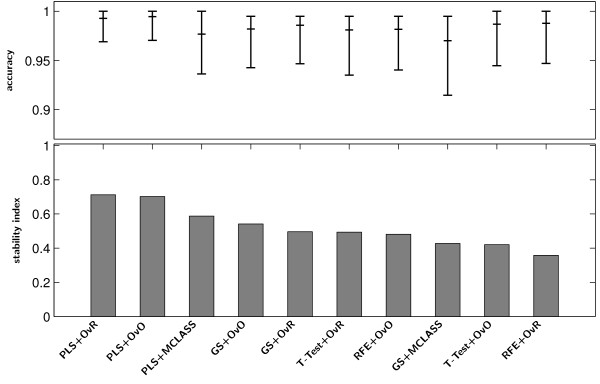
**Stability index *s***_***2***_** (bar chart) and accuracy of classification (dot chart) with the 95% confidence interval of the best classifier on the tested feature selection methods for MLL data.**

Figure 6 Stability index ***s***_***2***_(bar chart) and accuracy of classification (dot chart) with the 95% confidence interval of the best classifier on the tested feature selection methods for MLL data.

**Figure 7:**
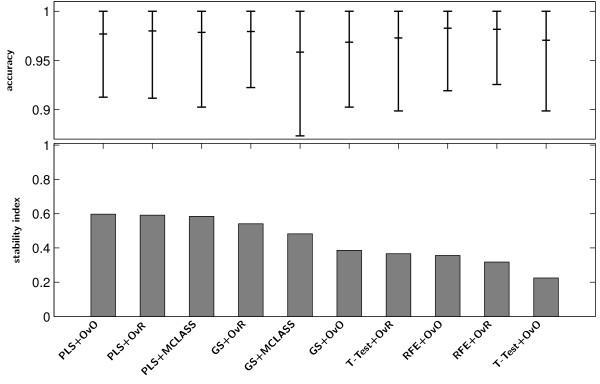
**Stability index *s***_***2***_** (bar chart) and accuracy of classification (dot chart) with the 95% confidence interval of the best classifier on the tested feature selection methods for SRBCT data.**

Figure 7 Stability index *s*_2_(bar chart) and accuracy of classification (dot chart) with the 95% confidence interval of the best classifier on the tested feature selection methods for SRBCT data.

**Figure 8:**
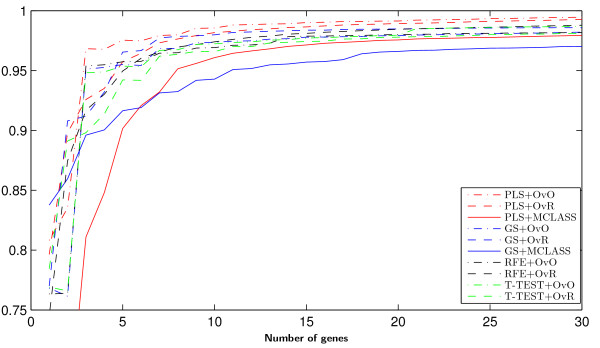
Accuracy of classification obtained by successive gene set reduction selected with all feature selection methods of the best classifier for MLL data.

Figure 8 Accuracy of classification obtained by successive gene set reduction selected with all feature selection methods of the best classifier for MLL data.

**Figure 9:**
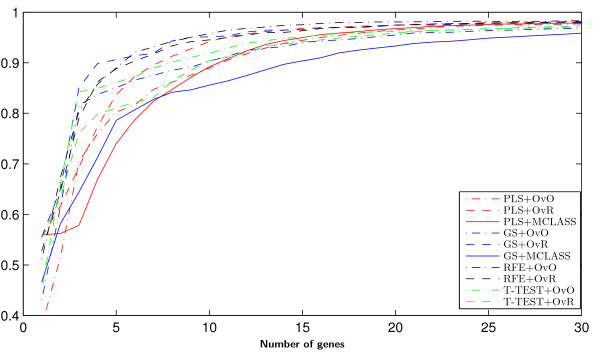
Accuracy of classification obtained by successive gene set reduction selected with all feature selection methods of the best classifier for SRBCT data.

Figure 9 Accuracy of classification obtained by successive gene set reduction selected with all feature selection methods of the best classifier for SRBCT data.

**Figure 10:**
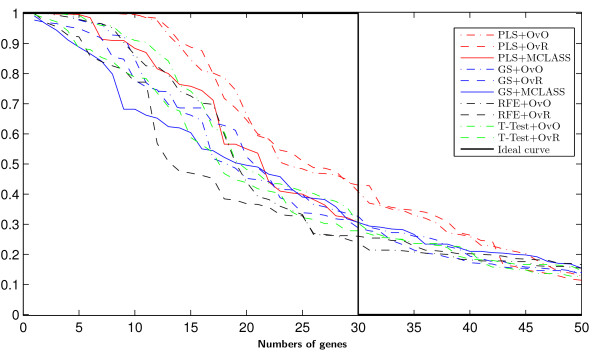
**Results of bootstrap-based feature ranking (BBFR) for the first 50 genes for MLL data.** In the ideal case (when gene lists are perfectly reproducible) the BBFR score reaches a value of 1 for the first selected genes and 0 for the rest (black curve).

Figure 10 Results of bootstrap-based feature ranking (BBFR) for the first 50 genes for MLL data. In the ideal case (when gene lists are perfectly reproducible) the BBFR score reaches a value of 1 for the first selected genes and 0 for the rest (black curve).

**Figure 11:**
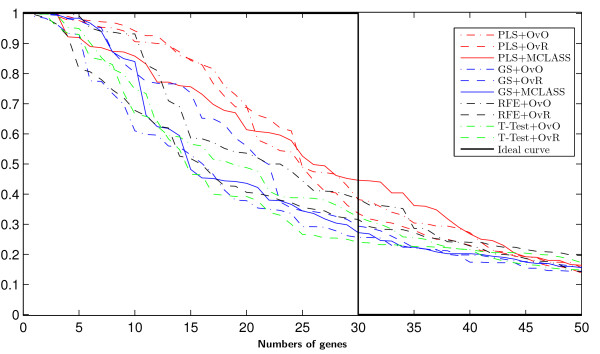
**Results of bootstrap-based feature ranking (BBFR) for the first 50 genes for SRBCT data.** In the ideal case (when gene lists are perfectly reproducible) the BBFR score reaches a value of 1 for the first selected genes and 0 for the rest (black curve).

Figure 11 Results of bootstrap-based feature ranking (BBFR) for the first 50 genes for SRBCT data. In the ideal case (when gene lists are perfectly reproducible) the BBFR score reaches a value of 1 for the first selected genes and 0 for the rest (black curve).

**Figure 12:**
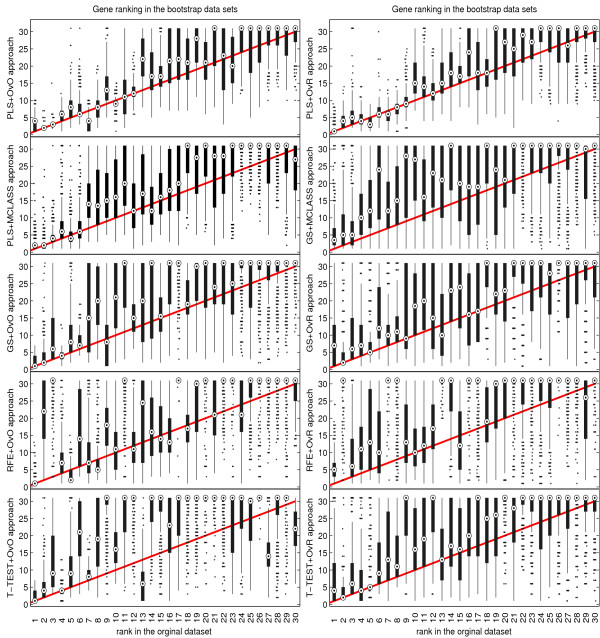
Comparison of rank boxplots in the bootstrap samples against rank in the original data set on all tested methods for MLL data.

Figure 12 Comparison of rank boxplots in the bootstrap samples against rank in the original data set on all tested methods for MLL data.

**Figure 13:**
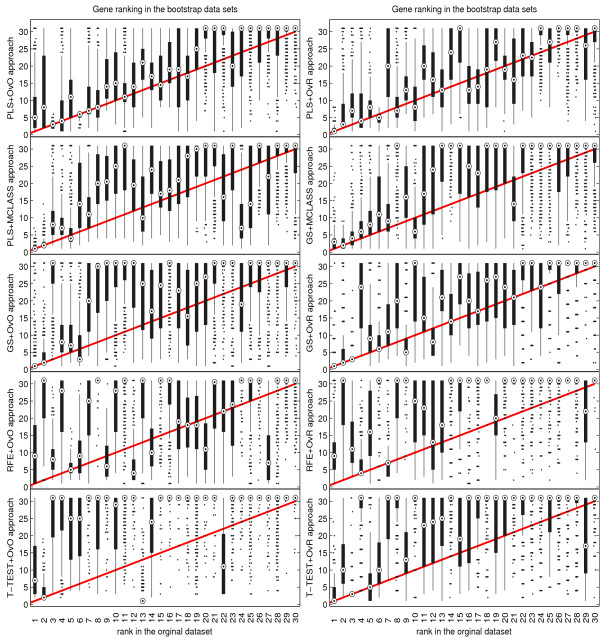
Comparison of rank boxplots in the bootstrap samples against rank in the original data set on all tested methods for SRBCT data.

Figure 13 Comparison of rank boxplots in the bootstrap samples against rank in the original data set on all tested methods for SRBCT data.

## Reviewer’s report 1

### Prof Marek Kimmel

### Report form

As the authors state, “gene expression profiles are a promising alternative for clinical cancer classification”. The well-known difficulty is the large dimension of the vector of data, compared to the usually modest number of independent data replicates. The authors propose a new, arguably better combination of known methods to face the classification problem. This is important; however, the most interesting problem tackled in the paper in a novel way is that of stability. Ranked gene lists can be unstable in the sense that a small change of the data set leads to serious changes in the resulting ordered lists. The authors address this issue by comparing how different methods yield different stability of results. Eventually, they find a new strategy to find a small subset of significant genes giving very stable feature rankings compared to currently employed methods. The paper seems interesting and suitable for Biology Direct. On the editorial side, some language usages are uncommon and therefore not clearly understandable, such as for example “invariability” which might mean “invariance” or “absence of variability”. I suggest using Oxford English Dictionary Online or a similar source to rectify these ambiguities (or employing a human text editor fluent in scientific English).

### Quality of written English

Needs some language corrections before being published

***Author’s response****We have edited the text and corrected the paper’s language mistakes.*

### Dr Hans Binder

### Report form

The manuscript Stable feature selection and classification algorithms for multiclass microarray data by Sebastian Student and Krzysztof Fujarewicz presents a new feature selection and multi-classification algorithm based on Partial Least Squares and decomposition into separate two-class problems. The authors clearly show that their method outperforms a series of state-of-the-art methods using appropriate benchmarks. The issue addressed is very important for the analysis of high-dimensional data and interesting for a broader readership as addressed by BD. Referencing and relation to state-of-the art is given appropriately. The method presented is novel, original and sound and obviously improves available solutions. Presentation, however, in general is suboptimal and requires revision. Particularly, I suggest the following points: 1. A large number of abbreviations are used and the reader gets completely lost in this jungle. I suggest to add a glossary which decodes and partly explains all abbreviations used, especially the different variants of methods used.

***Author’s response****We have added short subsection in theMethodsMethods section named: Symbols and abbreviations.*

2. The methodical part mixes basal points (e.g. how works PLS) with more peripheral ones (e.g. different benchmarking criteria such as stability plots etc.). The reader is overloaded with algorithmic details and formulae. The latter points are of course also important but many things become clear always on an intuitive level. I suggest to remove all non-essential details (e.g. all or, at least, part of the benchmark criteria) from the methodical part and to shift them into an appendix or supplementary text. The basal idea for benchmarks can be given in the methodical part very shortly in prosaic form (i.e. without formulae and algorithmic details).

***Author’s response****According to the suggestion we shift the part of the benchmark criteria into theAppendixsection.*

3. In my opinion, the methodical part should focus on the kernel of the new method, i.e. PLS and the decomposition into two-class comparisons and comparison with state of the art. Partly this information is given but mostly hidden in a heap of other things (see point 2.). A schematic figure that explains the essentials and novel aspects of the method and also visualizes the workflow might be very helpful. Possibly this scheme might visualize also differences with respect to other approaches. This point represents a real challenge but possibly the authors can solve it.

***Author’s response****We have added a new figure with a scheme that explains the gene selection method based on PLS.*

4. The authors used 3 data sets for verification and 4 types of presentation which provides 3x4=12 figures at the end. This broad data basis allows proper verification of the methods. However, the results of benchmarking of the three different data sets are mostly similar if not identical with respect to the benchmark criteria applied. Here the reader is overloaded with very similar figures with mostly redundant information content. I suggest removing 2/3 of the figures into a supplementary file and to show only one of each type in the main paper.

***Author’s response****We have moved figures for MLL and SRBCT datasets into aAppendixsection.*

5. In exceptional cases the results for the different data sets slightly differ (e.g. Figure 5 versus Figure 6). These details should be discussed.

***Author’s response****We have added short discussion about these results in theResults and Discussionsection*

6. The data sets are described in the Results-section, which dilutes the information content of the paper. I suggest moving this information into a ‘Data’-subsection in the Methods-chapter (incl. links and preprocessing).

***Author’s response****We have moved these data sets description into theDatasetssection.*

7. Main point: The benchmarking demonstrates that the PLS-variants used outperform the other methods. It would be desirable to understand the underlying principal reason for this difference and to generalize this finding. In the Conclusions section this question is shortly addressed. However, this issue, in my opinion, requires much more attention beyond all the benchmarking details. Obviously the decomposition of the multiclass problem into a series of two-class problems is more favorable than to solve the multiclass-problem at once. What is the deeper reason that causes this benefit. On the other hand: why, for example, simple t-testing performs worst. I strongly encourage the authors to extend the paper in this respect.

***Author’s response****We have extended theConclusionssection and have discussed these questions, but we must agree, that it is hard to find the real explanation for our findings, especially, because multiclass problems are more complicated, than the two class problems.*

8. The authors should provide a computer program of their approach along with the paper that might be used by others.

***Author’s response****Because bootstrap technique is computationally expensive, we apply our software on the computer cluster, which make it very difficult to publish. The main problem is that our software is not dedicated for personal computers, and for that reason we decided not to publish this code.*

Further minor points: 9. Both axes in all figures must be assigned. I.e. the y-axes must be labeled in Figures 5, 6 and 7 with ‘accuracy’ and in Figures 8, 9 and 10 with something else (BBFR-score which defines simply the mean degree of agreement of gene ranking after bootstrap).10. The step-function in Figures 8, 9 and 10 must be shortly explained in the legend and in the text (might I overlooked details). The ideal curve is far different from the real ones. The authors should discuss why a list-length of 30 was assumed. This choice seems rather arbitrary.11. Legend of Figures 8, 9 and 10: It is claimed that ‘every dot represents one gene’. I miss the dots.12. Please indicate that the Tables are provided in the supplement and not in the main text.13. Define accuracy on p. 3.14. Define BSS/WSS on p. 6 (sum of squares…???)!15. ‘Scalars’ should be presumably substituted by ‘scores’ on p. 7, line below Eq. (10)?

***Author’s response****We have edited the manuscript and corrected these mistakes.*

### Quality of written English

Acceptable

### Dr Yuriy Gusev

### Report form

General comments: The manuscript addresses one of the important problems in gene expression analysis i.e. feature selection for multiclass classicization of microarray data in cancer. While this problem has been investigated by many over past 10 years or so, the importance of utilization of gene expression data for classification of cancer samples remains high. This is mainly because of several potentially important practical applications in cancer diagnostics and prediction of drug response. Also – practical utilization of gene expression signatures has been questioned by many due to the known problems of reproducibility and validation. This paper addresses some of these issues by detailed analysis of stability of existing most popular classification algorithms as well as new method proposed by the authors. This study might have other important implications as it could be applicable to other types of global molecular profiling that are becoming more popular in recent years such as DNA copy number variation, exome profiling and RNAseq. The paper could benefit from additional discussion of this issue of applicability of the proposed methods for other types of omics data such as RNAseq and CNV.

***Author’s response****We have added in theConclusionssection the information about other possible applications of the presented feature selection methods.*

Strengths and weaknesses: This study has several strengths: a comparison of performance of many existing classification methods both in term of accuracy and stability of feature selection for the multiclass analysis. Also – this work is focused on developing of new methodology of effective identification of the most informative genes with main goal of finding a small subset of most accurate features. The authors for the first time have demonstrated effectiveness of decomposition of mutli-class classification problem into series of sub-problem of two-class selection. The important part of this work was applying these methods for analysis of 3 independent data set for 3 types of cancer. Weaknesses of this study include: throughout the study the authors rely on bootstrap resampling for all estimations of accuracy and stability which is quite common technique. However the validity of such approach for testing of gene expression classifiers has been questioned in the literature. It has been reported that permutation based estimates could be a poor substitute for testing a classier on an independent set of real gene expression data. It would be interesting to see how well the proposed methods perform when tested on such independent gene expression datasets. It would be good to see additional discussion of this issue in the paper.

***Author’s response****We have added additional discussion of the mentioned issue in the Bootstrap resampling section.*

Also – this study is using 500 resampling iterations for all steps of classifier construction however it is not clear if this is sufficient to ensure stability of the results, it would be useful if author could include additional comments on the reason for using 500 interactions.

***Author’s response****We have added additional explanation in the Bootstrap resampling section.*

Overall, this is detailed study of the important issues related to classification of cancer samples based on global gene expression profiling. It is addresses several technical issues of accuracy and stability of classification results. Reviewer recommends considering publishing this paper in more specialized journal which could provide a better targeted readership in the bioinformatics community.

***Author’s response****Because of applicability of the proposed methods for other high dimension biological data, this problem is important not only for readership in the bioinformatics. In our opinion the problems of reproducibility and stability of obtained features is especially important for biologists, people who work with biological data.*

### Quality of written English

Acceptable

## Competing interests

The authors declare that they have no competing interests.

## Authors’ contributions

SS and KF contributed equally to this work. Both authors read and approved the final manuscript.

## References

[B1] He Z Yu W Stable feature selection for biomarker discovery Comput Biol and Chem 2010 34 4 215 225 [http://arxiv.org/abs/1001.0887] 10.1016/j.compbiolchem.2010.07.002 20702140

[B2] Binder H Krohn K Burden CJ Washing scaling of GeneChip microarray expression BMC Bioinf 2010 11 291 [http://www.pubmedcentral.nih.gov/articlerender.fcgi?artid=2901370&tool=pmcentrez&rendertype=abstract] 10.1186/1471-2105-11-291 PMC2901370 20509934

[B3] Binder H Preibisch S Berger H Calibration of microarray gene-expression data Methods In Mol Biol Clifton Nj 2010 576 16 375 407 [http://www.ncbi.nlm.nih.gov/pubmed/19882273] 10.1007/978-1-59745-545-9_20 19882273

[B4] Dutkowski J Gambin A On consensus biomarker selection BMC Bioinf 2007 8 Suppl 5 S5 [http://www.ncbi.nlm.nih.gov/pubmed/17570864] 10.1186/1471-2105-8-S5-S5 PMC1892093 17570864

[B5] Zhang T Li C Ogihara M A comparative study of feature selection and multiclass classification methods for tissue classification based on gene expression Bioinformatics 2004 20 15 2429 2437 10.1093/bioinformatics/bth267 15087314

[B6] Draminski M Rada-Iglesias A Enroth S Wadelius C Koronacki J Komorowski J Monte Carlo feature selection for supervised classification Bioinformatics 2008 24 110 117 [http://www.bioinformatics.oxfordjournals.org/cgi/doi/10.1093/bioinformatics/btm486] 10.1093/bioinformatics/btm486 18048398

[B7] Guyon I Weston J Barnhill S Vapnik V Gene selection for cancer classification using support vector machines Machine Learning 2002 46 389 422 10.1023/A:1012487302797

[B8] Nguyen DV Rocke DM Tumor classification by partial least squares using microarray gene expression data Bioinformatics 2002 18 1 39 50 10.1093/bioinformatics/18.1.39 11836210

[B9] Höskuldsson A PLS regression methods J Chemom 1988 2 3 211 228 10.1002/cem.1180020306

[B10] Yang K Cai Z Li J Lin G A stable gene selection in microarray data analysis BMC Bioinf 2006 7 228 10.1186/1471-2105-7-228 PMC1524991 16643657

[B11] Gutkin M Dror G Shamir1 R SlimPLS: A method for feature selection in gene expression-based disease classification PLoS One 2009 4 7 10.1371/journal.pone.0006416 PMC2715895 19649265

[B12] Boulesteix A Slawski M Stability and aggregation of ranked gene lists Brief Bioinform 2009 10 5 556 568 10.1093/bib/bbp034 19679825

[B13] Boulesteix A Strobl C Augustin T Daumer M Evaluating Microarray-based classifiers: an overview Cancer Informatics 2008 6 77 97 19259405 10.4137/cin.s408 PMC2623308

[B14] Efron B Bootstrap methods: another look look at the jackknife Ann Stat 1979 7 1 26 10.1214/aos/1176344552

[B15] Braga-Neto U Dougherty ER Is cross-validation valid for small-sample microarray classification? Bioinformatics 2004 20 3 374 380 10.1093/bioinformatics/btg419 14960464

[B16] Garcia-Bilbao A Armananzas R Ispizua Z Calvo B Alonso-Varona A Inza I Larranaga P Lopez Vivanco G Suarez-Merino B Betanzos M Identification of a biomarker panel for colorectal cancer diagnosis BMC Cancer 2012 12 43 [http://www.ncbi.nlm.nih.gov/pubmed/22280244] 10.1186/1471-2407-12-43 22280244 PMC3323359

[B17] Abraham G Kowalczyk A Loi S Haviv I Zobel J Prediction of breast cancer prognosis using gene set statistics provides signature stability and biological context BMC Bioinformatics 2010 11 277 [http://www.pubmedcentral.nih.gov/articlerender.fcgi?artid=2895626&tool=pmcentrez&rendertype=abstract] 10.1186/1471-2105-11-277 20500821 PMC2895626

[B18] Armañanzas R Inza IN Larrañaga P Detecting reliable gene interactions by a hierarchy of Bayesian network classifiers Comput Methods Programs Biomed 2008 91 2 110 121 [http://www.ncbi.nlm.nih.gov/pubmed/18433926] 10.1016/j.cmpb.2008.02.010 18433926

[B19] Fu WJ Carroll RJ Wang S Estimating misclassification error with small samples via bootstrap cross-validation Bioinformatics 2005 21 9 1979 1986 [http://www.ncbi.nlm.nih.gov/pubmed/15691862] 10.1093/bioinformatics/bti294 15691862

[B20] Meuwissen TH Goddard ME Bootstrapping of gene-expression data improves and controls the false discovery rate of differentially expressed genes Genet Sel evol GSE 2004 36 2 191 205 10.1186/1297-9686-36-2-191 PMC2697185 15040898

[B21] Efron N Intrator N The effect of noisy bootstrapping on the robustness of supervised classification of gene expression data 2004 [http://ieeexplore.ieee.org/lpdocs/epic03/wrapper.htm?arnumber=1423002]

[B22] Dvison A Hinkley D Schechtman E Efficient bootstrap simulation Biometrika 1986 73 3 555 566

[B23] Hall P Performance of balanced bootstrap resampling in distribution function and Quantile problems Probability Theory 1990 85 239 260 10.1007/BF01277983

[B24] Efron B Tibshirani R Improvements on cross-validation: the 632+ bootstrap method J Amer Statist Assoc 1997 92 548 560

[B25] Efron B Estimating the error rate of a prediction rule: improvement on cross-validation J Amer Stat Assoc 1983 78 382 316 331 [http://www.jstor.org/stable/2288636?origin=crossref] 10.1080/01621459.1983.10477973

[B26] Wold H Soft modeling: the basic design and some extensions Syst Under Indirect Observation 1982 2 589 591

[B27] Tibshirani R Regression shrinkage and selection via the lasso J R Stat Soc Ser B Methodological 1996 58 267 288 [http://www.jstor.org/stable/2346178]

[B28] Hoerl AE Application of ridge analysis to regression problems Chem Eng Prog 1962 58 54 59

[B29] Chong IG Jun CH Performance of some variable selection methods when multicollinearity is present Chemom Intell Lab Systs 2005 78 1-2 103 112 [http://dx.doi.org/10.1016/j.chemolab.2004.12.011] 10.1016/j.chemolab.2004.12.011

[B30] Nguyen DV Rocke DM Multi-class cancer classification via partial least squares with gene expression profiles Bioinformatics 2002 18 9 1216 1226 [http://www.bioinformatics.oupjournals.org/cgi/doi/10.1093/bioinformatics/18.9.1216] 10.1093/bioinformatics/18.9.1216 12217913

[B31] Boulesteix AL PLS dimension reduction for classification with microarray data Stat Appl Genet Mol Biol 2004 3 Article33 [http://www.ncbi.nlm.nih.gov/pubmed/17049027] 10.2202/1544-6115.1075 16646813

[B32] Gidskehaug L Anderssen E Flatberg A Alsberg BK A framework for significance analysis of gene expression data using dimension reduction methods BMC Bioinf 2007 8 346+ [http://dx.doi.org/10.1186/1471-2105-8-346] 10.1186/1471-2105-8-346 PMC2194745 17877799

[B33] Johansson D Lindgren P Berglund A A multivariate approach applied to microarray data for identification of genes with cell cycle-coupled transcription Bioinformatics 2003 19 4 467 473 [http://www.bioinformatics.oupjournals.org/cgi/doi/10.1093/bioinformatics/btg017] 10.1093/bioinformatics/btg017 12611801

[B34] Martens H Modified Jack-knife estimation of parameter uncertainty in bilinear modelling by partial least squares regression (PLSR) Food Quality Preference 2000 11 1-2 5 16 [http://linkinghub.elsevier.com/retrieve/pii/S0950329399000397] 10.1016/S0950-3293(99)00039-7

[B35] Boulesteix AL Strimmer K Partial least squares: a versatile tool for the analysis of high-dimensional genomic data Briefings Bioinf 2007 8 32 44 [http://www.ncbi.nlm.nih.gov/pubmed/16772269] 10.1093/bib/bbl016 16772269

[B36] Musumarra G Barresi V Condorelli DF Fortuna CG Scirč S Potentialities of multivariate approaches in genome-based cancer research: identification of candidate genes for new diagnostics by PLS discriminant analysis J Chemom 2004 18 34 125 132 [http://doi.wiley.com/10.1002/cem.846] 10.1002/cem.846

[B37] Barker M Rayens W Partial least squares for discrimination J Chemom 2003 17 3 166 173 [http://doi.wiley.com/10.1002/cem.785] 10.1002/cem.785

[B38] Helland IS On the structure of partial least squares regression Commun Stat Simul Comput 1988 17 2 581 607 [http://www.informaworld.com/openurl?genre=article&doi=10.1080/03610918808812681&magic=crossref] 10.1080/03610918808812681

[B39] Geladi P Kowalski BR Partial least-squares regresion: a tutorial Analytica Chimica Acta 1986 185 1 17

[B40] De Jong S SIMPLS: An alternative approach to partial least squares regression Chemometrics Intell Lab Syst 1993 18 25 263

[B41] He H Jazdzewski K Li W Liyanarachchi S Nagy R Volinia S Calin GA Liu Cg Franssila K Suster S The role of microRNA genes in papillary thyroid carcinoma Proc Nat Acad Sci USA 2005 102 52 19075 19080 [http://www.pubmedcentral.nih.gov/articlerender.fcgi?artid=1323209&tool=pmcentrez&rendertype=abstract] 10.1073/pnas.0509603102 16365291 PMC1323209

[B42] VanStaveren WCG Solis DW Delys L Duprez L Andry G Franc B Thomas G Libert F Dumont JE Detours V Human thyroid tumor cell lines derived from different tumor types present a common dedifferentiated phenotype Cancer Res 2007 67 17 8113 8120 [http://www.ncbi.nlm.nih.gov/pubmed/17804723] 10.1158/0008-5472.CAN-06-4026 17804723

[B43] Zhang T Li C Ogihara M Evaluating reproducibility of differential expression discoveries in microarray studies by considering correlated molecular changes Bioinformatics 2009 25 13 1662 1668 10.1093/bioinformatics/btp295 19417058 PMC2940240

[B44] Fujarewicz K A multigene approach to differentiate papillary thyroid carcinoma from benign lesions: gene selection using bootstrap-based Support Vector Machines Endocrine - Related Cancer 2007 14 809 826 10.1677/ERC-06-0048 17914110 PMC2216417

[B45] Bhattacharjee Aaa Classification of human lung carcinomas by mRNA expression profiling reveals distinct adenocarcinoma subclasses PNAS 2001 98 24 13790 13795 10.1073/pnas.191502998 11707567 PMC61120

[B46] Ferrari F Bortoluzzi S Coppe A Sirota A Safran Maa Novel definition files for human GeneChips based on GeneAnnot BMC Bioinf 2007 8 446 10.1186/1471-2105-8-446 PMC2216044 18005434

[B47] Armstrong S Staunton J Silverman L Pieters R den Boer M Minden M Sallan S Lander E Golub T Korsmeyer S MLL translocations specify a distinct gene expression profile that distinguishes a unique leukemia Nat Genet 2002 30 41 47 10.1038/ng765 11731795

[B48] Khan J Wei J Ringner M Saal L Ladanyi M Westermann F Berthold F Schwab M Antonescu C Peterson C Meltzer P Classification and diagnostic prediction of cancers using gene expression profiling and artificial neural networks Nat Med 2001 7 673 679 10.1038/89044 11385503 PMC1282521

[B49] Detours V Wattel S Venet D Hutsebaut N Bogdanova T Tronko MD Dumont JE Franc B Thomas G Maenhaut C Absence of a specific radiation signature in post-Chernobyl thyroid cancers British J Cancer 2005 92 8 1545 1552 [http://www.pubmedcentral.nih.gov/articlerender.fcgi?artid=2362019&tool=pmcentrez&rendertype=abstract] 10.1038/sj.bjc.6602521 PMC2362019 15812549

[B50] Jarzab B Wiench M Fujarewicz K Simek K Jarzab M Oczko-Wojciechowska M Wloch J Czarniecka A Chmielik E Lange D Gene expression profile of papillary thyroid cancer: sources of variability and diagnostic implications Cancer Res 2005 65 4 1587 1597 [http://www.ncbi.nlm.nih.gov/pubmed/15735049] 10.1158/0008-5472.CAN-04-3078 15735049

[B51] Fujarewicz K Kimmel M Rzeszowska-Wolny J Swierniak A A note on classification of gene expression data using support vector machines J Biol Syst 2003 11 43 56 10.1142/S0218339003000658

